# The effect of the rate of hydrostatic pressure depressurization on cells in culture

**DOI:** 10.1371/journal.pone.0189890

**Published:** 2018-01-09

**Authors:** Ellen Tworkoski, Matthew R. Glucksberg, Mark Johnson

**Affiliations:** 1 Department of Biomedical Engineering, Northwestern University, Evanston, Illinois, United States of America; 2 Department of Mechanical Engineering, Northwestern University, Evanston, Illinois, United States of America; 3 Department of Ophthalmology, Northwestern University, Chicago, Illinois, United States of America; Duke University, UNITED STATES

## Abstract

Changes in hydrostatic pressure, at levels as low as 10 mm Hg, have been reported in some studies to alter cell function in vitro; however, other studies have found no detectable changes using similar methodologies. We here investigate the hypothesis that the rate of depressurization, rather than elevated hydrostatic pressure itself, may be responsible for these reported changes. Hydrostatic pressure (100 mm Hg above atmospheric pressure) was applied to bovine aortic endothelial cells (BAECs) and PC12 neuronal cells using pressurized gas for periods ranging from 3 hours to 9 days, and then the system was either slowly (~30 minutes) or rapidly (~5 seconds) depressurized. Cell viability, apoptosis, proliferation, and F-actin distribution were then assayed. Our results did not show significant differences between rapidly and slowly depressurized cells that would explain differences previously reported in the literature. Moreover, we found no detectable effect of elevated hydrostatic pressure (with slow depressurization) on any measured variables. Our results do not confirm the findings of other groups that modest increases in hydrostatic pressure affect cell function, but we are not able to explain their findings.

## Introduction

Cells are continuously exposed to a range of mechanical stresses due to the dynamic nature of the biological environment in which they reside. It has been recognized that some of these physical stimuli can be sensed by cells and play a significant role in influencing cell signaling and behavior. Stretch-activated ion channels, membrane-bound enzymes, and internal cytoskeletal filaments have all been shown to respond to compressive, tensile, and shear stresses [1]. Over the last two decades, a number of studies have also reported significant changes in cell behavior following the application of hydrostatic pressure in the range of 10–150 mm Hg to cells cultured in vitro on rigid substrates [2–31]. These changes include increases in cell proliferation and migration, increases in apoptosis, changes in cell morphology, and changes in gene expression. As biological cells and tissues are essentially incompressible [32], application of hydrostatic pressure will generate insignificant mechanical strain in these cells, and thus it is surprising that hydrostatic pressure would have any effect on them.

It is possible that when hydrostatic pressure is applied to the cells, artifacts are introduced that affect cell function. Indeed, Lei et al. [33] showed that when hydrostatic pressure is applied by use of a hydrostatic fluid column, hypoxic conditions are created that alter cell function. Once the effects of hypoxia were controlled for, no effect of hydrostatic pressure on cell behavior was observed in these studies. Other methods of application of hydrostatic pressure, such as use of a pressurized chamber [2,5,11,13,26,34] and use of a pump-driven flow system [6,15,21] are not subject to this hypoxia artifact. Use of a pressurized chamber alters the gas composition in equilibrium with the culture medium [35], but the magnitude of these changes are small for modest changes in hydrostatic pressure.

Agar et al. [11] proposed that application of hydrostatic pressure to a cell would necessarily involve transient changes in pressure during the initial pressurization step and the final depressurization step, and these transients might affect the cells. Resta et al. [36] provided data supporting this expectation. The objective of our study was to determine if the rate by which the system is depressurized, following application of hydrostatic pressure, might have an effect on cells in culture and potentially be responsible for the observed effects that had been previously attributed to hydrostatic pressure. We tested this hypothesis by replicating hydrostatic pressure experiments already reported in the literature [5,11,21,26] on bovine aortic endothelial cells (BAECs) and a PC12 neuronal cell line, while also examining the effects of a slow and rapid rate of depressurization of the system after application of hydrostatic pressure for various time periods, as compared with controls exposed to atmospheric pressure. The time periods chosen and variables assayed were based on the experiments already reported in the literature.

## Methods

### Cell culture

Bovine aortic endothelial cells (B304-05) and bovine endothelial cell growth medium (B211-500) were purchased from Cell Applications. Media was changed every other day. Cells were passaged upon reaching ~80% confluency and split 1:3–1:6. Endothelial cells of passages 3–8 were used for all experiments.

Rat adrenal pheochromocytoma PC12 cells [37] were purchased from ATCC. Undifferentiated PC12 cells were initially grown in suspension in growth media: RPMI-1640 Medium (ATCC) supplemented with 10% heat-inactivated horse serum (Life Technologies), 5% fetal bovine serum (Atlanta Biologicals), and 1% penicillin-streptomycin (Fisher Scientific). Fresh media was added to the flasks every 2–3 days. Cells were passaged every 4–6 days and split 1:3–1:5. To induce cell attachment, PC12 cells were seeded in plates coated with type IV collagen (Sigma). Following plating, cells were left in growth media overnight and then differentiated to a neuronal phenotype. To induce differentiation, PC12 cells were cultured in RPMI-1640 medium supplemented with 1% heat-inactivated horse serum, 1% penicillin-streptomycin, and 50 ng/ml nerve growth factor (NGF) 2.5S (Life Technologies) for 3 days prior to assaying [11].

### Pressure chambers

**Fig 1:**
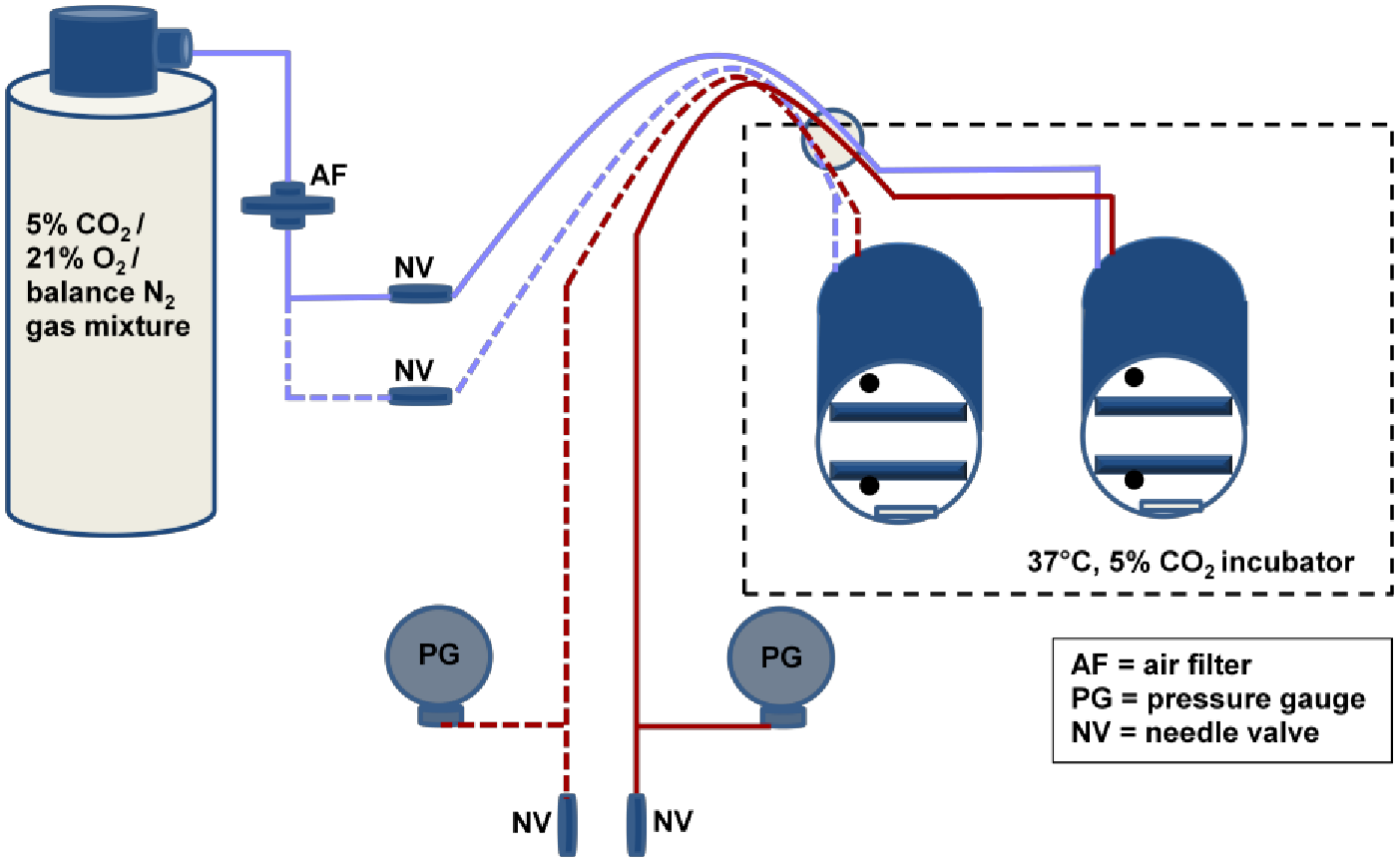
Schematic of the pressure chamber set-up. Chambers depicted with the lids off. Lids were tightly screwed into place and sealed with a silicone O-ring prior to pressurization. Tubing colored in blue represents the inlet gas streams while tubing colored in red represents the outlet gas streams. Dotted vs. solid colored lines are used to distinguish tubing attached to the left vs. right pressure chambers respectively. The two black dots near the top and bottom of each pressure chamber represent the positions of the outlet and inlet gas streams, respectively. The two large rectangles within each chamber represent the shelves used to hold cell culture plates, while the smaller rectangles at the bottoms of each chamber represent the water reservoirs. The dotted black line represents the 37°C incubator.

Two aluminum cylindrical pressure chambers (5” inner diameter, 6” length, 0.5” thick) were designed to fit inside a standard 37°C, 5% CO_2_, 95% air incubator (Fig 1). Two shelves were fitted inside each chamber to support cell culture plates. Water reservoirs were placed at the base of each chamber two to three days prior to the start of experiments to create a humidified atmosphere. Temperature and humidity were measured inside each chamber using a remote humidity/temperature monitor (VWR) and confirmed to match that within the incubator (37°C, 95% relative humidity). Water levels in the water baths were checked after each experiment and every other day for multi-day experiments. Neither the water level nor the media levels covering the cells were noticeably depleted for any experiment. A separate experiment showed humidity levels were constant and matched incubator conditions for multi-day experiments.

A tank containing a 5% CO_2_/21% O_2_/balance N_2_ gas mixture (Airgas) was placed just outside the incubator and fitted with a 2-stage, ultra-low pressure delivery regulator (Airgas). Polyurethane hose (1/8” ID, Clippard) was used to feed the gas mixture into and out of the pressure chambers through a port at the back of the incubator. A 0.2 μm inline air filter (Thermo Scientific) was plumbed into the inlet circuit. Digital pressure gauges (Ashcroft, DG25; 0.5% of 0-15psi span accuracy, +/- 1 mm Hg sensitivity; relative to atmospheric pressure) were included in the outlet circuit and used to monitor the chamber pressure levels. Miniature needle valves (Clippard) were placed into the inlet and outlet circuits of each chamber to provide tight control over the gas flow. A slow, steady gas flow rate (~0.1 ml/min or ~0.005% of the chamber volume/min) through each chamber was maintained during each experiment to help maintain pressure and allow air flow during multi-day experiments.

### Experimental procedure

Cells were plated and cultured prior to pressurization in an assay-dependent manner, specified below. Two plates were placed inside the pressure chambers (one into each chamber) while a third plate containing control cells to be cultured under atmospheric pressure was placed inside the incubator, but outside of the pressure chambers themselves. A gage pressure of 100 mm Hg was set within both chambers for all experiments. The designation of each chamber as the “slow depressurized” or “rapid depressurized” chamber was randomly assigned at the start of each experiment.

Depending on the experiment, elevated hydrostatic pressure was applied anywhere from 3 hrs to 9 days. For all experiments that ran longer than 24 hrs, chambers were depressurized every other day to allow for media changes. The appropriate depressurization rate, depending on the experimental group, was used for each of these depressurizations. “Rapid” chambers were depressurized over ~5 seconds while “slow” chambers were depressurized steadily over 30 minutes. The former rate was picked within the range of rates found in the literature (e.g. [11,19, 20]), while the latter was picked to be much larger than this value.

The chamber pressures were checked periodically for fluctuations during each experiment. If any fluctuations were noted, they were first compared to any changes that occurred in the ambient atmospheric pressure that was monitored using a barometer (H-B Instrument, accuracy 2.6 mm Hg). If the change in chamber pressures (relative to atmospheric pressure) were due to the change in atmospheric pressure, no alterations were made to the system. This ensured that the absolute pressure level in each of the chambers was held constant. However, if the chambers experienced an increase or decrease in pressure that did not correspond to a change in the atmospheric pressure, the gage pressure was manually adjusted back to 100 mm Hg above the initial atmospheric pressure. Variations of up to +/- 7 mm Hg (< 1% of the absolute pressure applied) were seen in chamber pressure.

### Cell viability assays

Following a 3 hour, 24 hour, 2 day, or 4 day application of elevated hydrostatic pressure, BAEC viability was visualized using a LIVE/DEAD viability kit for mammalian cells (Life Technologies, L3224). BAECs were rinsed and stained with 4 μM ethidium homodimer-1, and 2 μM calcein AM in a phosphate buffered saline (PBS) solution for 15 min at 37°C. Cells were then rinsed and observed under a Leica DMIRB inverted fluorescence microscope using ex 488 nm/em 515 nm for calcein detection and ex 530 nm/em 617 nm for ethidium homodimer-1 detection.

BAEC and PC12 cell viability after elevated hydrostatic pressure treatment was also assessed in some experiments using CellTox Green Cytotoxicity Assay (Promega). BAECs were plated in 96 well plates at a density of 31,250 cells per cm^2^. CellTox dye was added to the cell suspension in a 1:500 dilution as per the manufacturer’s instructions at the time of plating. BAECs were given 24 hrs to attach, and then hydrostatic pressure was applied for 3 hours. CellTox fluorescence was measured at various times (0, 2, 4, 8.5, 22.5 hr) following depressurization using a Molecular Devices Gemini EM fluorescence plate reader (ex 485nm/em 532nm). PC12 cells were plated in collagen-coated 96 well plates at a density of 10,000 cells/cm^2^ [11] and differentiated for 3 days. On the third day of differentiation, CellTox dye was added in a 1:500 dilution along with fresh differentiation media and incubated with the cells for 24 hrs. Hydrostatic pressure was then applied for 3 hours and CellTox fluorescence was measured at 0, 2, 4, 8, and 24 hr timepoints following depressurization.

In all cell viability and cell apoptosis (next section) studies, a positive control was done by treating a separate group of cells cultured under ambient pressure with staurosporine (1 μM in a 0.1% DMSO in PBS solution created from a 1mM DMSO stock solution), a compound that is known to inhibit a range of protein kinases and trigger apoptosis, for 24 hrs prior to measurement; a vehicle control of 0.1% DMSO was also used. In the case of CellTox viability experiments, staurosporine was added 24 hrs prior to the first measurements of cell viability and remained in the wells for the duration of the experiment.

### Apoptosis assays

BAEC and PC12 apoptosis levels were quantified using an Annexin V-FITC (BD Pharmingen) assay. For Annexin V staining, cells were seeded at a density of 10,000 cells/cm^2^ and cultured to 80–90% confluency in 6 well plates. They were then subjected to elevated hydrostatic pressure for 3 hrs. Either immediately after depressurization (BAECs), or after a 24 hr recovery period (PC12 cells), cells were detached using Accutase (BD Biosciences), centrifuged for 6 min (BAECS) or 10 min (PC12 cells) at 200xg, and the pellet resuspended in 100 μl 1X Annexin V binding buffer. PC12 cell resuspension required gentle pipetting with a 26-gauge needle in order to properly disperse cell clumps. Cells were then transferred into 5ml polystyrene round-bottom tubes (Corning Falcon, 12x75mm), stained with FITC-conjugated Annexin V that was diluted 1:20 into the suspension, and counter-stained with a membrane-impermeable 500 nM DAPI stain for 10 min on ice in the dark. 400 μl additional cold 1X Annexin V binding buffer was then added to each tube and samples were immediately passed through a BD LSRII benchtop flow cytometer. Four quadrants were superimposed over the resulting DAPI vs. Annexin V plots to distinguish between necrotic cells (DAPI positive/Annexin negative), late stage apoptotic cells (DAPI positive/Annexin positive), healthy cells (DAPI negative/Annexin negative), and early stage apoptotic cells (DAPI negative, Annexin positive). Quadrant boundaries were determined based on single stain or no stain samples as well as by ambient pressure negative controls, which contained primarily healthy cells, and staurosporine-treated positive controls, which contained primarily early or late stage apoptotic cells. Boundaries were set such that at least 85% of the ambient pressure negative control cells were within Q3 (Annexin negative/DAPI negative).

BAEC apoptosis was further examined using a TUNEL (Roche) assay. In preparation for TUNEL staining, BAECs were plated at a density of 31,250 cells/cm^2^ within 96 well plates (Corning 3603), and allowed to attach overnight. BAECs were then subjected to elevated hydrostatic pressure for 3 hrs. Immediately after depressurization the media was removed, and cells were fixed in a 4% paraformaldehyde PBS solution for 1 hr at room temperature. Cells were then rinsed once with PBS, and permeabilized with 0.1% Triton X-100 in a 0.1% sodium citrate solution in PBS for 5 min at 4°C. Cells were again rinsed with PBS, stained using the TUNEL reaction solution for 60 mins at 37°C, rinsed twice, and visualized using an Olympus IX83 inverted fluorescence microscope using a GFP filter (ex 488nm/em 525nm). A positive control was created by treating some ambient pressure wells with a 1000 units/ml DNase I recombinant solution in a 50 mM Tris buffer solution (pH 7.5, containing 1mg/ml bovine serum albumin) for 10 min at room temperature following cell permeabilization, but prior to staining. An additional ambient pressure negative control was created by staining fixed and permeabilized cells with labeling solution only (no terminal deoxynucleotidyl transferase).

### Proliferation assays

BAECs were plated at a seeding density of 2000 cells/cm^2^ in 6 well plates (Corning) and allowed to attach overnight. Elevated hydrostatic pressure was then applied for 9 days, with media changes being performed every other day. Cell counts were taken at 0, 1, 3, 5, 7, and 9 days. At each of the six time points, a single well from each of the 6 well plates (rapidly depressurized, slowly depressurized, atmospheric pressure negative control) was measured. Cells were detached with 0.25% Trypsin/EDTA, centrifuged (6min at 200xg), resuspended in media, and counted using a hemocytometer. Different wells from each plate were measured at each time point. The seeding density, pressure duration, and experimental protocol were adapted from Sumpio et al. [5].

BAEC proliferation was also measured using CyQUANT Cell Proliferation Assay Kit (Life Technologies). We found that the dye gave a fluorescent signal that was linear with cell density for the range of densities we examined (S1 Fig). BAECS were seeded at levels ranging from low subconfluency to confluency (densities of 3125; 7813; 15,625; and 31,250 cells/cm^2^) in 96 well plates (Costar), allowed to attach overnight, and then subjected to a 24 hr hydrostatic pressure treatment. After depressurization, all wells were rinsed once with PBS, all liquid was aspirated, and the plates were frozen at -80°C for 2 days. The plates were then thawed and incubated for 5 min at room temperature with a mixture of 1X lysis buffer and 2X CyQUANT GR dye. Fluorescence at a 485/530nm ex/em was measured using a Molecular Devices Gemini EM plate reader.

### Actin imaging

BAECs were plated in low walled 35 mm ibiTreat-coated dishes (ibidi 80136) and allowed to attach overnight prior to pressurization. Three different pressure durations and three different initial cell seeding densities were used: 3 hrs (27,429 cells/cm^2^), 24 hrs (21,428 cells/cm^2^), or 7 days (2000 cells/cm^2^). Media was changed every other day over the duration of the 7-day experiment. Following depressurization, cell media was aspirated, cells were rinsed twice with PBS, and cells were then immediately fixed in 4% paraformaldehyde PBS solution at room temperature for 10 mins. Each dish was washed twice with PBS, and then cells were permeabilized with 0.1% Triton X-100 solution for 5 mins at room temperature. Following two more PBS rinses, a PBS solution containing 1% bovine serum albumin (BSA) (Sigma) was added to each dish and incubated for 30 mins at room temperature to reduce non-specific background staining. Each dish was then rinsed and stained for 20 min at room temperature with a 165 nM solution of Alexa Fluor 488 phalloidin (Invitrogen) in PBS containing 1% BSA. Each dish was then rinsed and counterstained for 30 min at room temperature with a 2 ug/ml Hoechst 33342 trihydrochloride, trihydrate (Invitrogen) solution in PBS. The cells were then rinsed and imaged with an inverted Zeiss Axio Observer.Z1 confocal microscope equipped with a 2-photon laser.

### Statistical analysis

As the goal of these experiments was to replicate results of previous studies and determine if the rate of depressurization might play a causative role in these results, only findings that confirmed those of previous studies were examined for statistical significance; as such, only one-sided t-tests were applied. This statistical procedure, as opposed to examining all results and using two-sided t-tests, affected only a single result that is noted in the text, and that result would have no impact on the conclusions of this study. When multiple comparisons were made, a Bonferroni correction was applied such that the calculated p-value was determined as [1-(1-p*)^m^] where p* is the p-value for a single test, and m is the number of tests. Statistical comparisons for the viability/apoptosis assays and the 24 hr BAEC proliferation assay were made using unpaired Student’s t-tests with a significance level of 0.05. For the BAEC 9-day proliferation experiments, a multiple regression linear model was also used with cell count expressed as a function of three variables: day number, an indicator variable distinguishing between experimental and control groups, and a second indicator variable distinguishing between rapidly/slowly depressurized groups with a significance level of 0.05.

## Results

In BAECs, we examined the effects of hydrostatic pressure application and depressurization on cell viability, apoptosis levels, cell proliferation rates and intracellular actin distribution. In PC12 neuronal cells, the effects on cell viability and apoptosis levels were examined. The cell types and variables investigated were largely dictated by the published studies that we were comparing against (see Table 1).

**Table 1:** Summary of pressure treatments and results from the current study in comparison with results from similar experiments in the literature.

Assay	Cell Type	Current Study		Past Literature		
		Pressure treatment	Results	Pressure treatment	Results	Reference
**Cell Viability and Apoptosis**	Endothelial cells*	3 hrs, 100 mm Hg^1^	No increase in apoptosis following depressurization. No change in cell viability during 24 hrs following depressurization	12–48 hrs, 40 mm Hg^2^	Decrease in cell number / loss in confluence after 24 hrs	[6]
		2 d or 4 d, 100 mm Hg^1^	No change in cell viability following depressurization	2–6 d, 40, 80, 120, or 160 mm Hg^1^ on HUVECs**	Cell death after exposure to over 80 mm Hg for over 2 d	[2]
		--	--	1–9 d, 40, 80, or 120 mm Hg^1^	No change in cell viability	[5]
	***PC12 cells***	3 hrs, 100 mm Hg^1^	No change in cell viability during 24 hrs following depressurization	1 or 24 hrs of 15 or 70 mm Hg^1^	Increased apoptosis after 1 hr of 15 or 70 mm Hg. Apoptosis levels increased with increased time and pressure	[26]
		3 hrs, 100 mm Hg^1^	No increase in apoptosis 24 hrs after pressure initiation	2 hrs of 100 mm Hg^1^	Increased apoptosis 24 hrs after pressure initiation	[11]
**Cell Proliferation**	Endothelial cells*	24 hrs, 100 mm Hg^1^; variety of initial cell seeding densities	High seeding densities: no change. Low seeding density: decreased proliferation in fast depressurized cells compared to slow depressurized or negative controls	24 hrs of 50, 100, or 150 mm Hg^2^	Increased proliferation with increased pressure	[21]
		1–9 d, 100 mm Hg^1^	No change in proliferation following depressurization	1–9 d of 40, 80, or 120 mm Hg^1^	Increased proliferation in cells exposed to 120, 80, or 40 mm Hg after 3, 7, or 9 d, respectively	[5]
		--	--	2–6 d of 40, 80, 120, or 160 mm Hg^1^ on HUVECs**	Increased proliferation after 2 d. Decreased proliferation after 4–6 d	[2]
**Cell Morphology and Actin Distribution**	Endothelial cells*	3 hrs, 100 mm Hg^1^	No differences in cell morphology or actin distribution	24 hrs of 50, 100, or 150 mm Hg^2^	Cell elongation, increased centrally and peripherally located actin filaments, appearance of multilayers.	[21]
		24 hrs, 100 mm Hg^1^	No differences in cell morphology or actin distribution	24 hrs of 50, 100, or 150 mm Hg^2^	Cell elongation, development of thick centrally located stress fibers, bilayered structure	[15]
		7 d, 100 mm Hg^1^	No differences in cell morphology or actin distribution	1–9 d of 40, 80, or 120 mm Hg^1^	Cell elongation, random cell alignment, decrease in peripheral actin filaments, increase in central actin	[5]
		--	--	12–48 hrs of 40 mm Hg^2^	Cell rounding, protrusions appeared, increased number of central actin filaments, less prominent peripheral actin	[6]

*Bovine aortic endothelial cells (BAECs) unless otherwise noted.

**Human umbilical vein endothelial cells (HUVECs).

^1^ Pressurized chamber was used for pressure treatment.

^2^ Pump driven flow chamber was used for pressure treatment.

### BAEC viability studies

**Fig 2:**
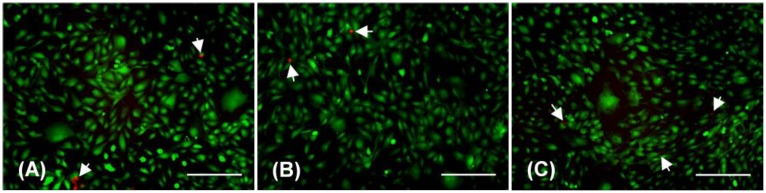
Fluorescent LIVE/DEAD images of BAECs after exposure to elevated hydrostatic pressure for 3 hours. Cells were depressurized by either rapid depressurization (A) or slow depressurization (B), or exposed only to ambient atmospheric pressure (negative control) (C), and then stained with LIVE/DEAD assay. Live cells are green; dead cells are stained red and indicated by the white arrows. Images represent data gathered from three replicates per experimental group. Each well was surveyed in its entirety and at least two representative images were taken for each experimental group. Scale Bar = 100 μm.

LIVE/DEAD assays performed immediately following application of hydrostatic pressure for 3 hours showed that the vast majority of cells in the rapidly depressurized, slowly depressurized, and negative control groups were viable with no obvious staining differences between the three groups (Fig 2). A few dead cells were observed in all samples.

**Fig 3:**
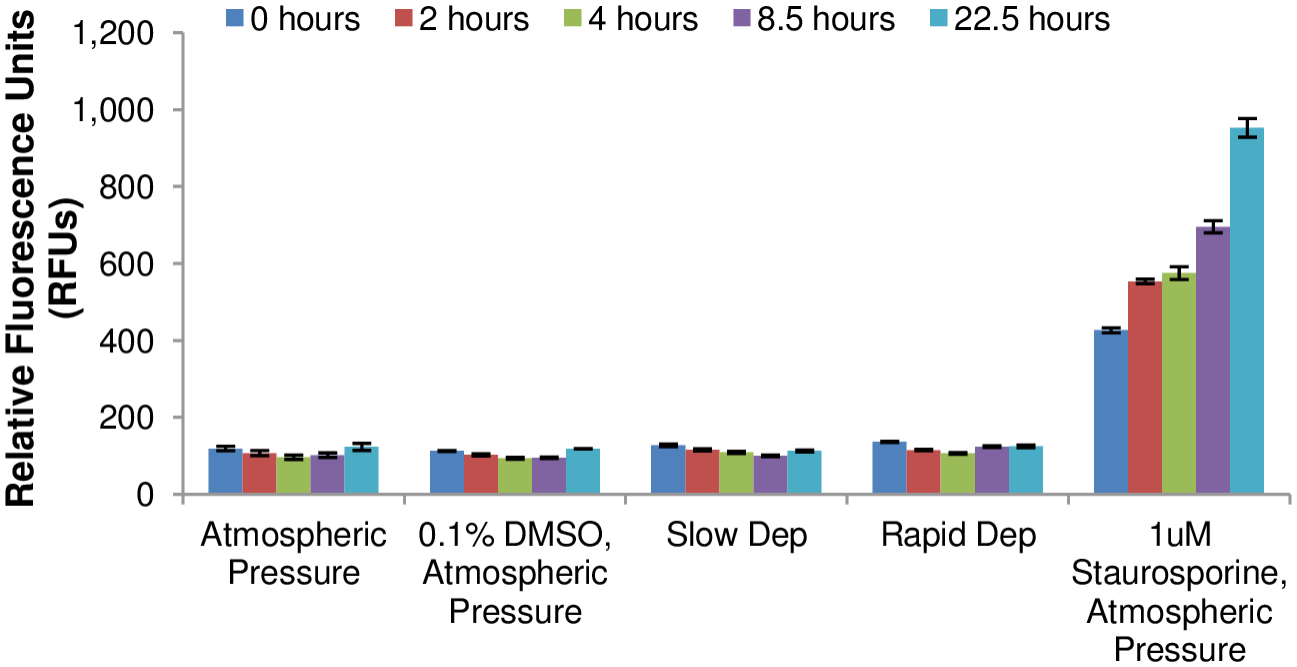
Level of BAEC death following pressurization as indicated by CellTox. Cell viability was monitored over the course of 24 hrs for an ambient atmospheric pressure negative control, a vehicle negative control (0.1% DMSO), two experimental groups that received a 100 mm Hg hydrostatic pressure treatment for 3 hrs and were then either slowly or rapidly depressurized, and finally a staurosporine treated (1μM for 24 hrs before measurements) positive control. Cell viability was monitored by measuring fluorescence on a plate reader (RFU = relative fluorescence units). Error bars represent SE. Data is the mean of three to five replicates per experimental group.

In order to determine if the pressurization treatment might have a delayed effect on BAEC viability, a CellTox assay was performed over a 24 hr period following depressurization. The results shown in Fig 3 indicate that there was no change in cell viability with time following the application of a hydrostatic pressure for 3 hrs, for either a rapid or slow depressurization. A negative, ambient atmospheric pressure control as well as a positive control treated with a known apoptosis-inducing agent, staurosporine, and a vehicle control of 0.1% DMSO are also shown.

**Fig 4:**
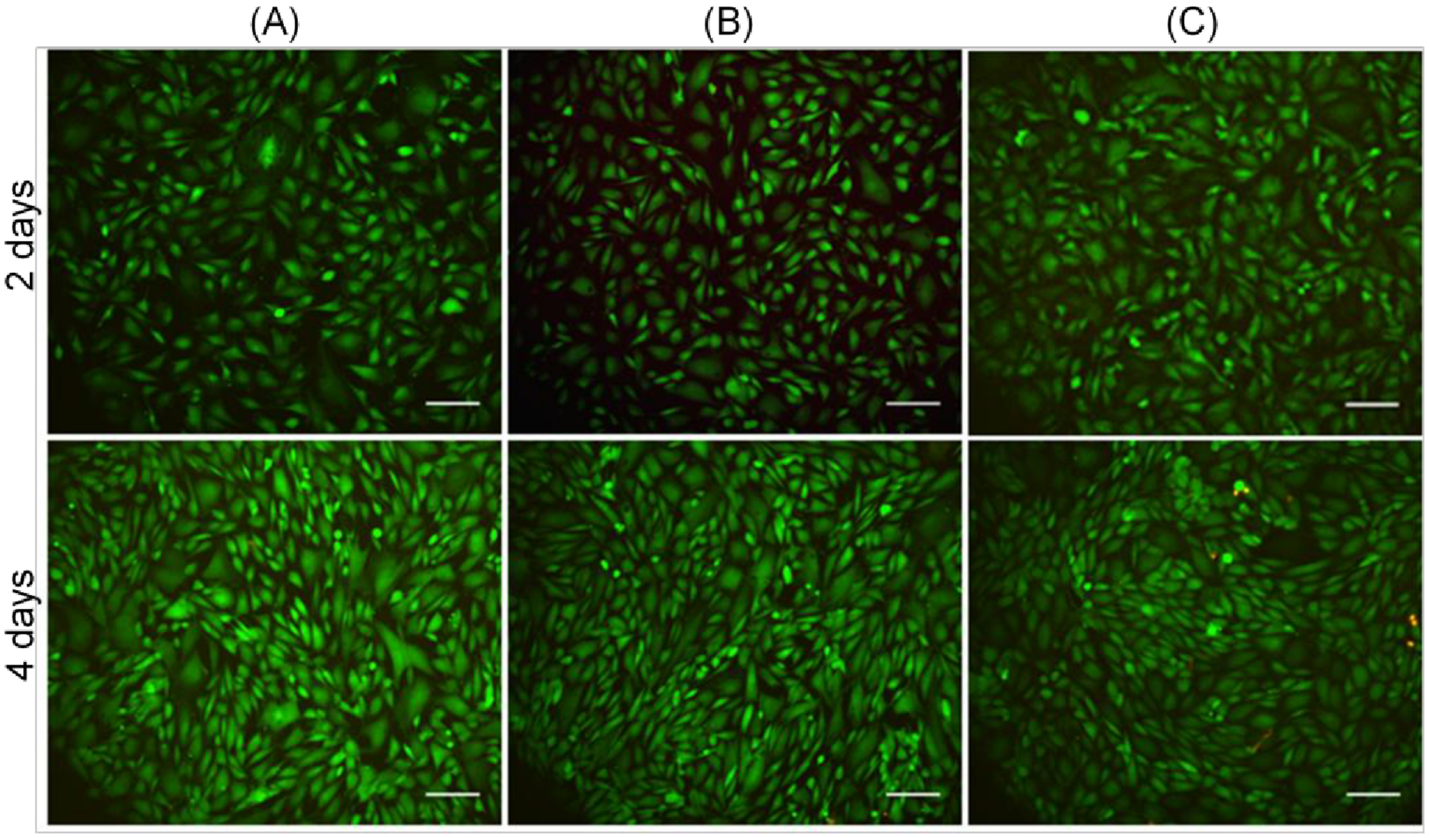
Fluorescent LIVE/DEAD images of BAECs after exposure to hydrostatic pressure for either 2 days or 4 days. Cells were depressurized by either rapid depressurization (A) or slow depressurization (B) or exposed only to ambient atmospheric pressure (negative control) (C) and then stained as in Fig 2. The vast majority of observed cells were live as indicated by the green stain. Scale Bar = 100 μm.

To determine if longer pressure durations would have an effect on BAEC viability, LIVE/DEAD assays were performed on cells after either 2 or 4 days of elevated hydrostatic pressure. The resulting fluorescent images, taken soon after the cells were depressurized, again showed no difference between the viability levels of cells depressurized rapidly, cells depressurized slowly, or control cells (Fig 4).

### BAEC apoptosis studies

**Fig 5:**
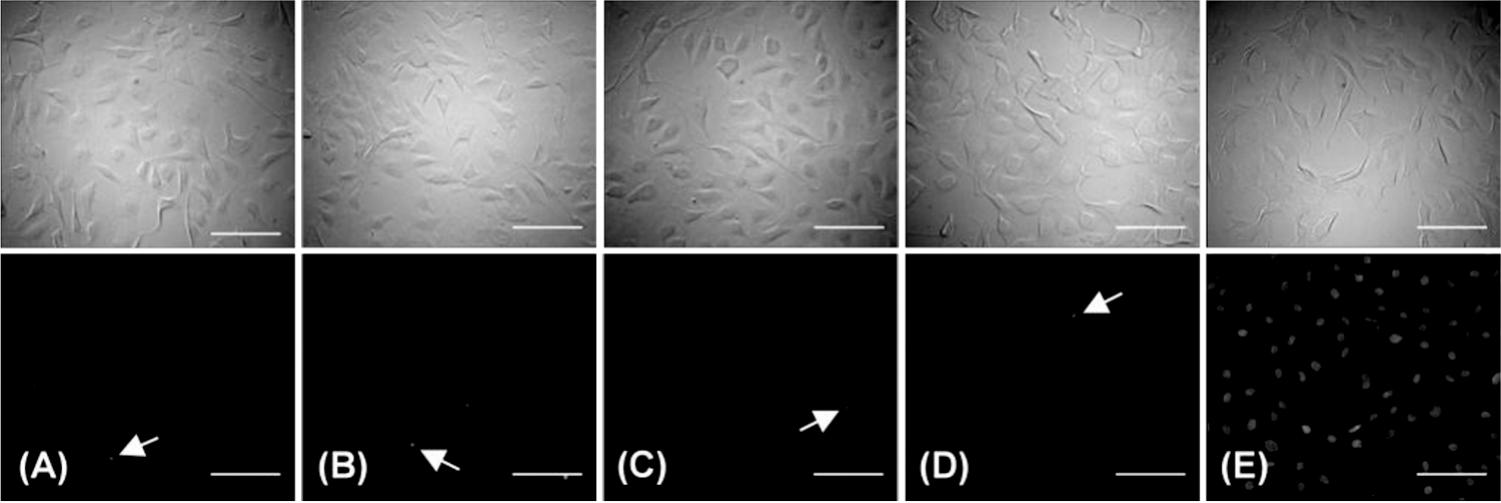
Differential interference contrast (top) and TUNEL stained fluorescent (bottom) images of BAECs. Images of cells immediately following rapid depressurization (A) or slow depressurization (B) after a 3 hr application of elevated hydrostatic pressure along with two negative controls (C: atmospheric pressure and D: atmospheric pressure without using terminal transferase) and one positive control (E: DNase recombinant I). White arrows indicate locations of TUNEL staining, a few of which were seen in panels A-D; panel E shows that nearly all of the positive control cells were apoptotic. The images represent data gathered from two replicates per experimental group. Scale bar = 100 μm.

Apoptotic levels in BAECs following application of hydrostatic pressure for 3 hrs were assessed immediately after either rapid or slow depressurization using TUNEL and Annexin V assays. Differential interference contrast (DIC) and TUNEL-stained fluorescence images are shown in Fig 5 for these cells, along with two negative controls and one positive control. The levels of apoptosis seen were negligible (Fig 5A and 5B) and comparable to a negative control exposed only to atmospheric pressure (Fig 5C), as well as a second negative control in which cells were only exposed to the labeling solution component of the TUNEL stain without the necessary transferase (Fig 5D). In contrast, the positive control showed extensive apoptosis (Fig 5E).

**Fig 6:**
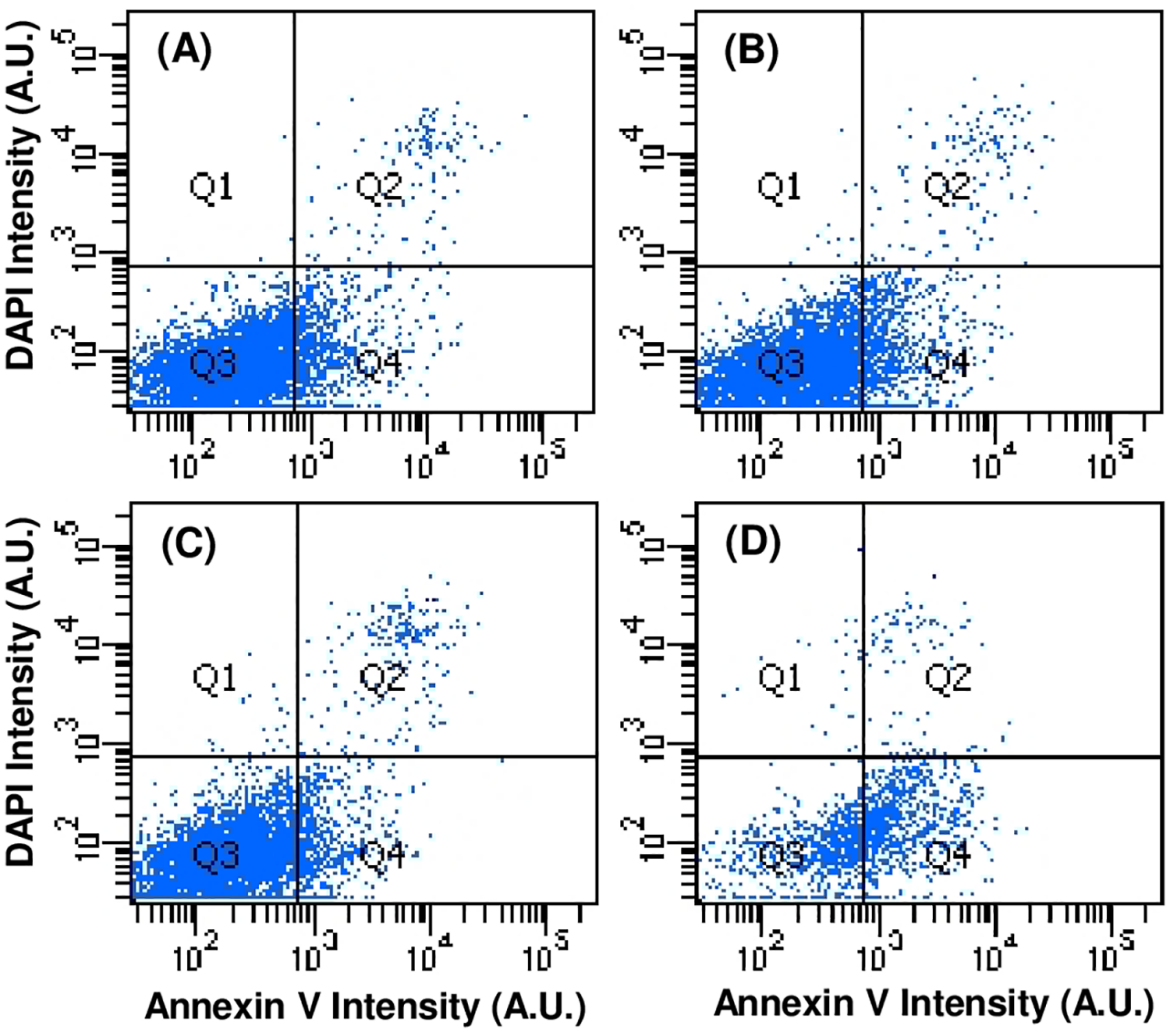
Flow cytometry data for BAECs stained with Annexin V and counterstained with DAPI. Staining was done immediately after subjecting cells to rapid depressurization (A) or slow depressurization (B) following a 3 hr application of elevated hydrostatic pressure. Results for a negative control (C: atmospheric pressure) and a positive control (D: treatment with 1 μM staurosporine for 24 hrs) are also shown. Annexin V and DAPI fluorescence intensity, in arbitrary units (A.U.), are given on the abscissa and ordinate, respectively. Cytometry data is presented as four quadrants superimposed over the resulting DAPI vs. Annexin V plots to distinguish between necrotic cells (DAPI positive/Annexin negative), late stage apoptotic cells (DAPI positive/Annexin positive), healthy cells (DAPI negative/Annexin negative), and early stage apoptotic cells (DAPI negative, Annexin positive).

Using flow cytometry, Annexin V and DAPI levels were measured in BAECs under these same conditions and confirmed the TUNEL results as shown in Fig 6 for the rapidly depressurized, slowly depressurized, ambient pressure negative control, and positive control staurosporine-treated groups. Table 2 summarizes the percentages of cells found in each quadrant for these four groups. Points in quadrants 1, 2, 3, and 4 correspond to dead necrotic cells, dead apoptotic cells, healthy cells, and early stage apoptotic cells respectively. The vast majority of cells in the first three groups (85–87%) were Annexin V negative and DAPI negative (Q3), indicating that they were healthy. No significant differences were seen between rapidly depressurized cells, slowly depressurized cells, or ambient pressure negative controls. Positive control staurosporine-treated cells, on the other hand, showed the expected indications of apoptosis.

**Table 2:** Percentages of BAEC populations in each of the four Annexin V/DAPI quadrants indicated in the flow cytometry plots in Fig 6.

Experimental Groups	Quadrants			
	*Q1 (dapi + annexin -)*	*Q2 (dapi + annexin +)*	*Q3 (dapi - annexin -)*	*Q4 (dapi - annexin +)*
*Negative control (atmospheric pressure)*	0.3%	3.1%	86.4%	10.1%
*Rapidly depressurized*	0.1%	1.7%	87.0%	11.3%
*Slowly depressurized*	0.2%	2.2%	85.0%	12.7%
*Positive control (1μM staurosporine*, *atmospheric pressure)*	1.2%	5.1%	43.9%	49.8%

### BAEC proliferation

**Fig 7:**
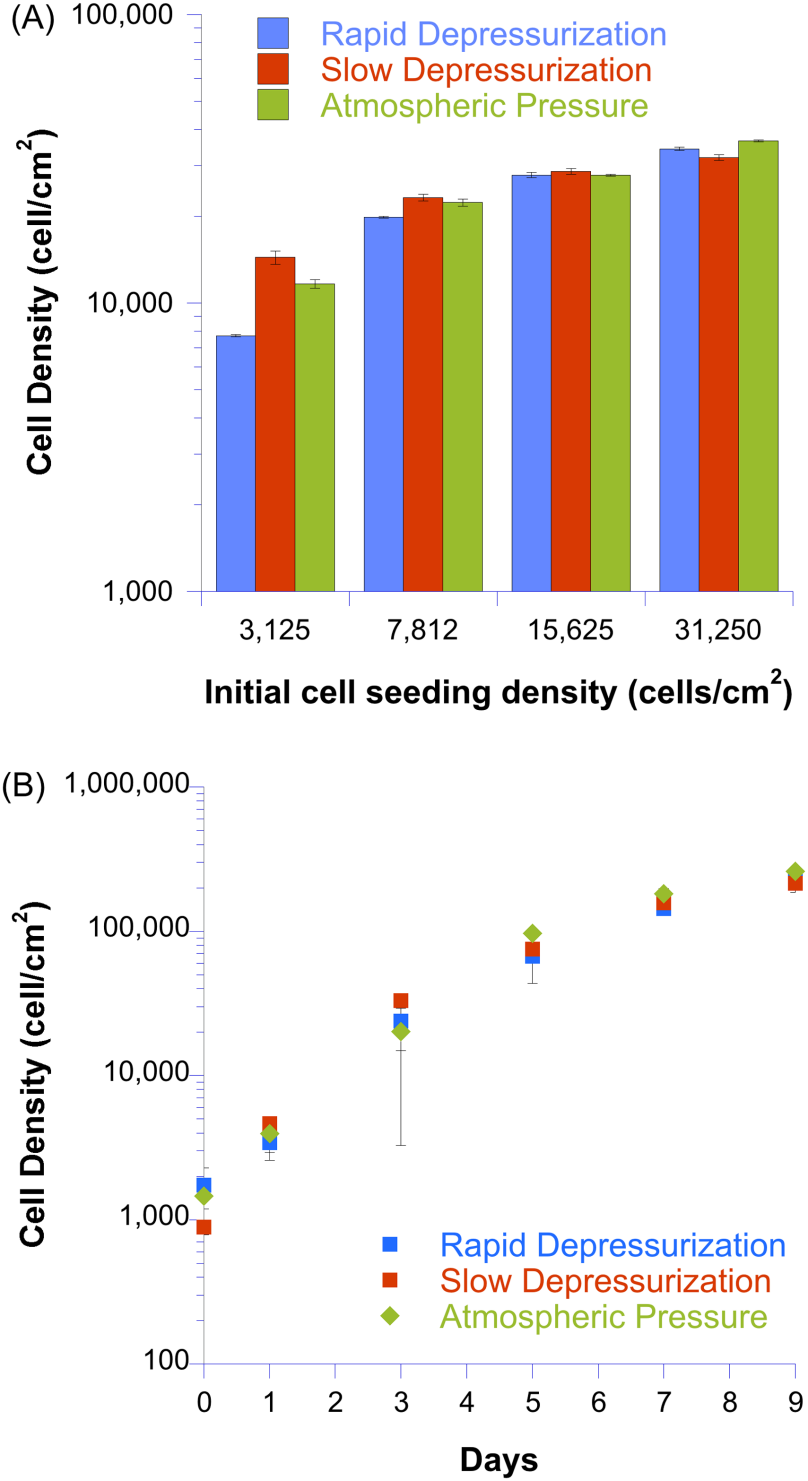
Cell proliferation assays for BAECs exposed to elevated hydrostatic pressure for indicated periods and then subjected to rapid depressurization or slow depressurization along with a negative control (atmospheric pressure). (A) CyQUANT assay results after 24 hr application of elevated hydrostatic pressure as a function of initial cell seeding density within single wells of a 96 multiwell plate. Data represents mean +/-SE for 3–5 replicates within each experimental group. (B) Cell counts within 6 well plates taken using a hemocytometer as a function of days exposed to elevated hydrostatic pressure. Data displayed is an average of two individual experimental runs (exception is the 9 day cell count point for the atmospheric pressure negative control data set; this represents a single run). Two to four cell counts were taken at each time for each run. Error bars represent standard error.

Cell proliferation studies were conducted on BAECs exposed to hydrostatic pressure for various periods and then subjected to rapid depressurization or slow depressurization. The cell densities obtained at four different initial cell seeding densities for cells exposed to hydrostatic pressure for 24 hours and then depressurized either rapidly or slowly as compared to ambient pressure negative control samples were measured using a fluorescent CyQUANT dye, and are shown in Fig 7A. The calibration curve used for the CyQUANT dye can be found in the supplemental material (S1 Fig). A series of one-sided unpaired t-tests were used to assess potential increases in proliferation in pressurized cells relative to negative control samples, as had been reported in the literature [21], at all four initial seeding densities. No significant increase in the final cell count was observed for rapidly depressurized or slowly depressurized BAECs relative to ambient pressure negative controls for any initial seeding density (Fig 7A: p > 0.8).

The results for an initial seeding of 3125 cells/cm^2^ require further comment. For this data set, the number of cells counted for the rapidly depressurized sample was significantly less than that counted for either the slow depressurized or negative control sample (p < 0.02 using a two-sided t-test). No significant differences were observed between the slow depressurized and negative control samples. This result is opposite from what is reported in literature (pressurized BAECS had a higher proliferation rate than control [5,21]), resulting in p > 0.99 for a one-sided t-test. Thus, our result does not confirm what was reported in the literature. Because of this result, additional experiments were conducted, one with 12 replicates and one with 60 replicates per experimental group (S2 Fig), that produced similar results (p < 0.01 using the two-sided t-test) to our first experiment. This result suggested a difference between rapid and slow depressurization of the cells, in agreement with our hypothesis. However, since we only saw this result for the low initial seeding density and not with higher seeding densities or longer pressurization times (see next paragraph) and because our result was in variance with the literature [21], we suspect that this is a statistical outlier and therefore chose not to highlight this finding.

In a second set of experiments, BAECs were cultured under elevated hydrostatic pressure for 9 days and, at 6 timepoints during this period, cell counts were taken using a hemocytometer. As shown in Fig 7B, very similar cell proliferation trends were observed for rapidly depressurized, slowly depressurized, and negative control groups. No obvious signs of cell death were observed upon visual inspection of all samples prior to cell counting at any time point. A multiple regression linear model was then fitted to the cell count data using the following relationship:

$$y=\alpha_{0}+\alpha_{1}x_{1}+\alpha_{12}x_{1}x_{2}$$

where y is the cell count, x_1_ is the day number, and x_2_ is an indicator variable distinguishing between ambient pressure (1) and elevated pressure (0) groups. The interaction term (*α*_*12*_) was not found to be statistically significant (p > 0.9), indicating no significant increase in cell count due to elevated pressure. Adding a third indicator variable distinguishing between rapid depressurized (1) and slow depressurized (0) groups was also examined and found to have no significant effect (p > 0.6). Thus, we found no effect of elevated hydrostatic pressure or depressurization rate on BAEC proliferation in contrast to previous findings [5,21].

### BAEC actin distribution

**Fig 8:**
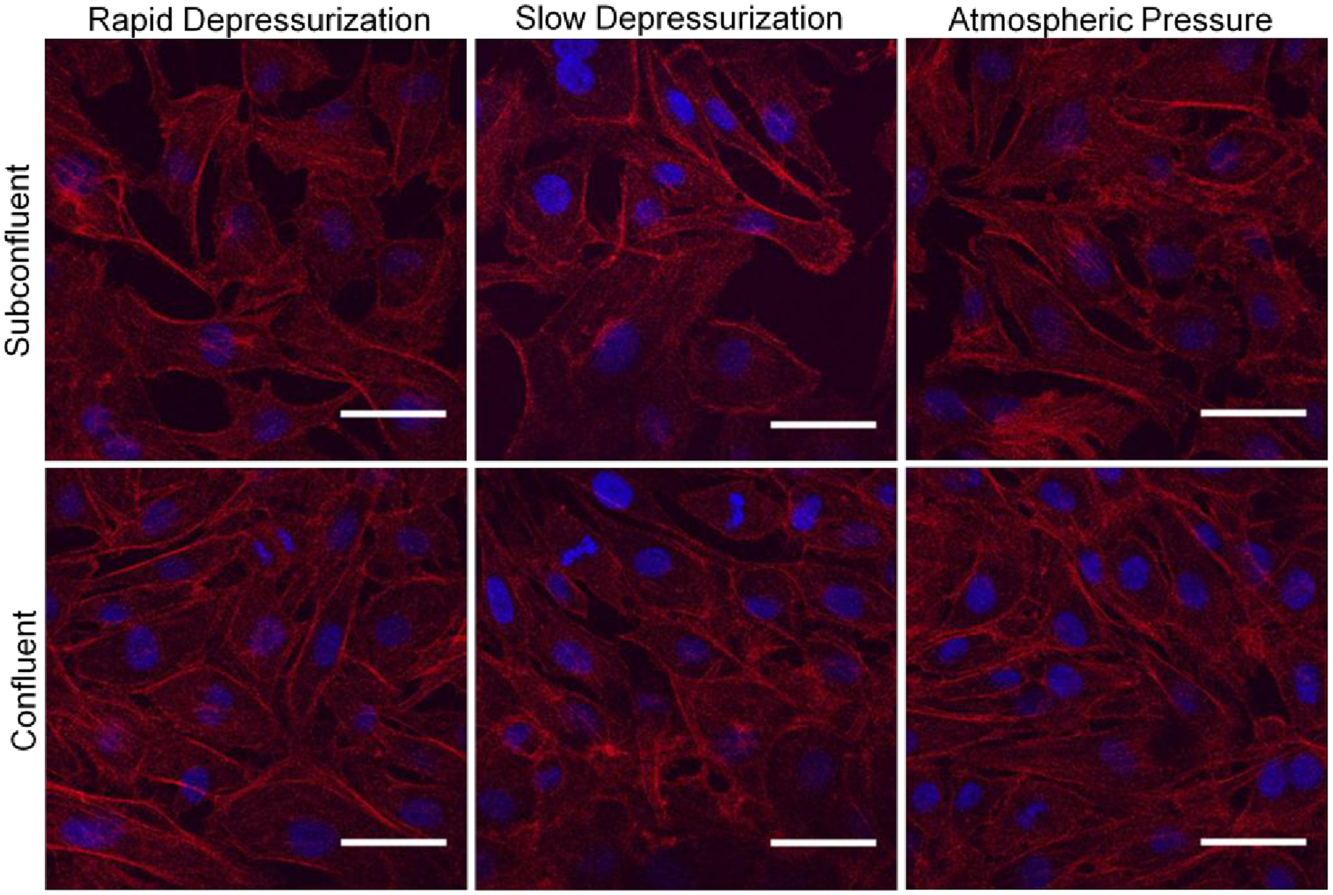
F-actin distribution in BAECs after 3 hrs of exposure to elevated hydrostatic pressure followed by rapid depressurization or slow depressurization as compared with a negative control (atmospheric pressure). F-actin is stained in red; nuclear DNA is stained in blue. Subconfluent regions of each sample are depicted in the top row while confluent regions of each sample are depicted in the bottom row. Scale bar = 40 μm.

BAECs were subjected to a hydrostatic pressure treatment for 3 hours followed by a rapid or slow depressurization, stained with phalloidin, and imaged. The morphology of both subconfluent and confluent cells subjected to elevated hydrostatic pressure could not be distinguished from control cells (Fig 8).

**Fig 9:**
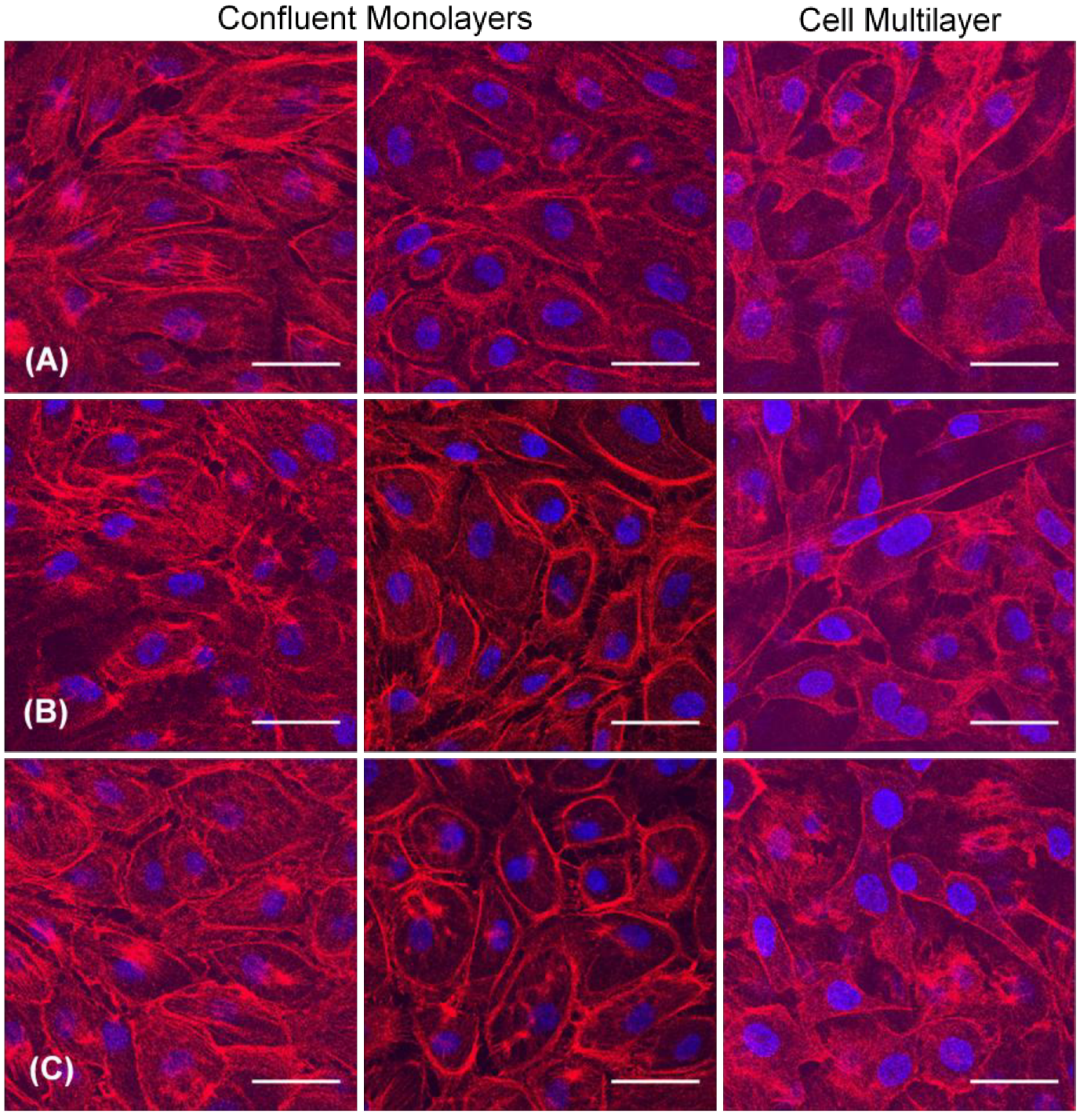
Confocal images of F-actin distribution in BAECs after exposure to elevated hydrostatic pressure for 24 hours followed by either rapid (A) or slow (B) depressurization as compared with a negative control (C: atmospheric pressure). F-actin is stained in red; nuclear DNA is stained in blue. The first two columns, which both contain images of confluent monolayers within each sample, were taken at z heights approximately 0.5–1 μm apart, moving from the surface (first column) down into the interior of the cell (second column). The third column contains images of cell multilayers which were found in all samples. Scale bar = 40 μm.

Pressurization treatments of longer duration yielded similar results. Examples of images from an experiment in which confluent BAECs were subjected to elevated hydrostatic pressure for 24 hours are shown in Fig 9. The confocal images shown there illustrate how variations in the z height of the focal plane can result in altered perceptions of intracellular actin distribution. When comparing images that had been taken at similar z heights, there were no notable differences in cell morphology or actin filament distribution between the three groups. The third column in Fig 9 illustrates that, depending on the region of the sample observed, some layers of overlapping cells (cell multilayering) could be observed for the negative control as well as for the pressurized samples.

**Fig 10:**
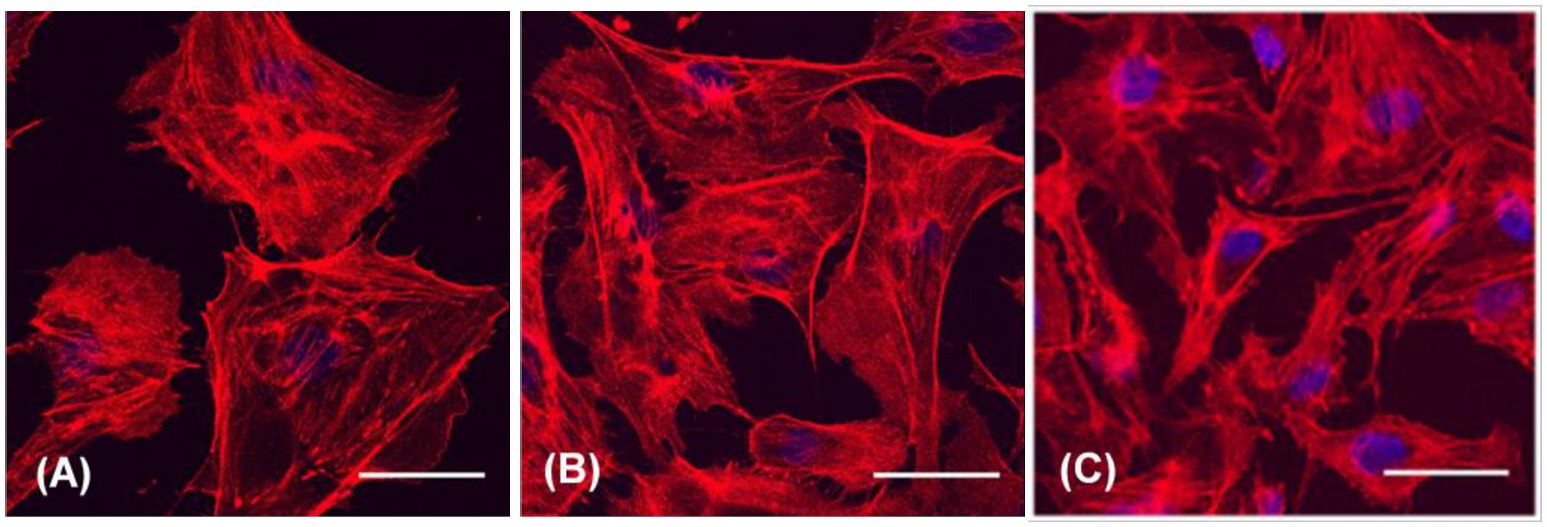
Confocal images of F-actin distribution in BAECs after exposure to elevated hydrostatic pressure for 7 days followed by either rapid (A) or slow (B) depressurization as compared with a negative control (C: atmospheric pressure). F-actin is stained in red; nuclear DNA is stained in blue. Scale bar = 40 μm.

Finally, BAECs were subjected to a 100 mm Hg pressure treatment for 7 days. Images of these cells again showed no apparent differences in actin filament distribution patterns and overall morphologies when comparing pressurized cells and cells that were cultured solely under ambient pressure (Fig 10). Cells in all three groups did show an increase in size compared to cells cultured for shorter time periods, possibly indicating cellular senescence.

### PC12 viability studies

**Fig 11:**
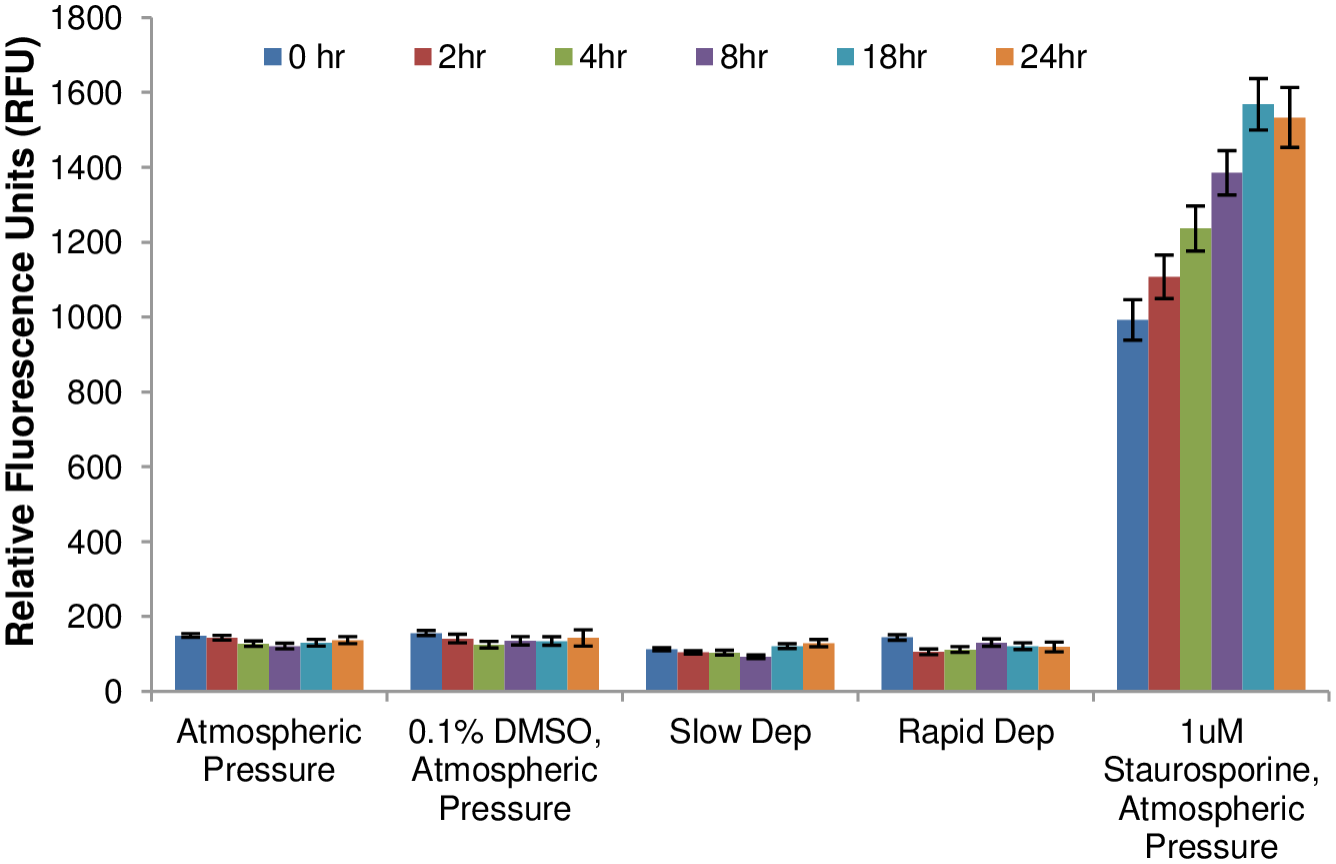
PC12 viability following exposure to elevated hydrostatic pressure for 3 hours, rapid or slow depressurization, and additional culture time from 0 to 24 hours. Comparisons with two negative controls (atmospheric pressure, and 0.1% DMSO vehicle controls) and positive control (1 μM staurosporine prior to the first timepoint) are shown. Cell death as measured by the CellTox assay is shown in relative fluorescence units (RFUs). Error bars represent standard error.

The viability of differentiated PC12 cells was tracked over a 24 hour period following exposure to elevated hydrostatic pressure using a CellTox assay. As shown in Fig 11, no differences in CellTox fluorescence levels were apparent in the pressurized cells compared to negative controls, nor was there a difference between cells depressurized rapidly versus those depressurized more slowly. A positive control group, which was treated with 1 μM staurosporine, showed significant cell death that increased with post-exposure time, as expected.

### PC12 apoptosis studies

**Fig 12:**
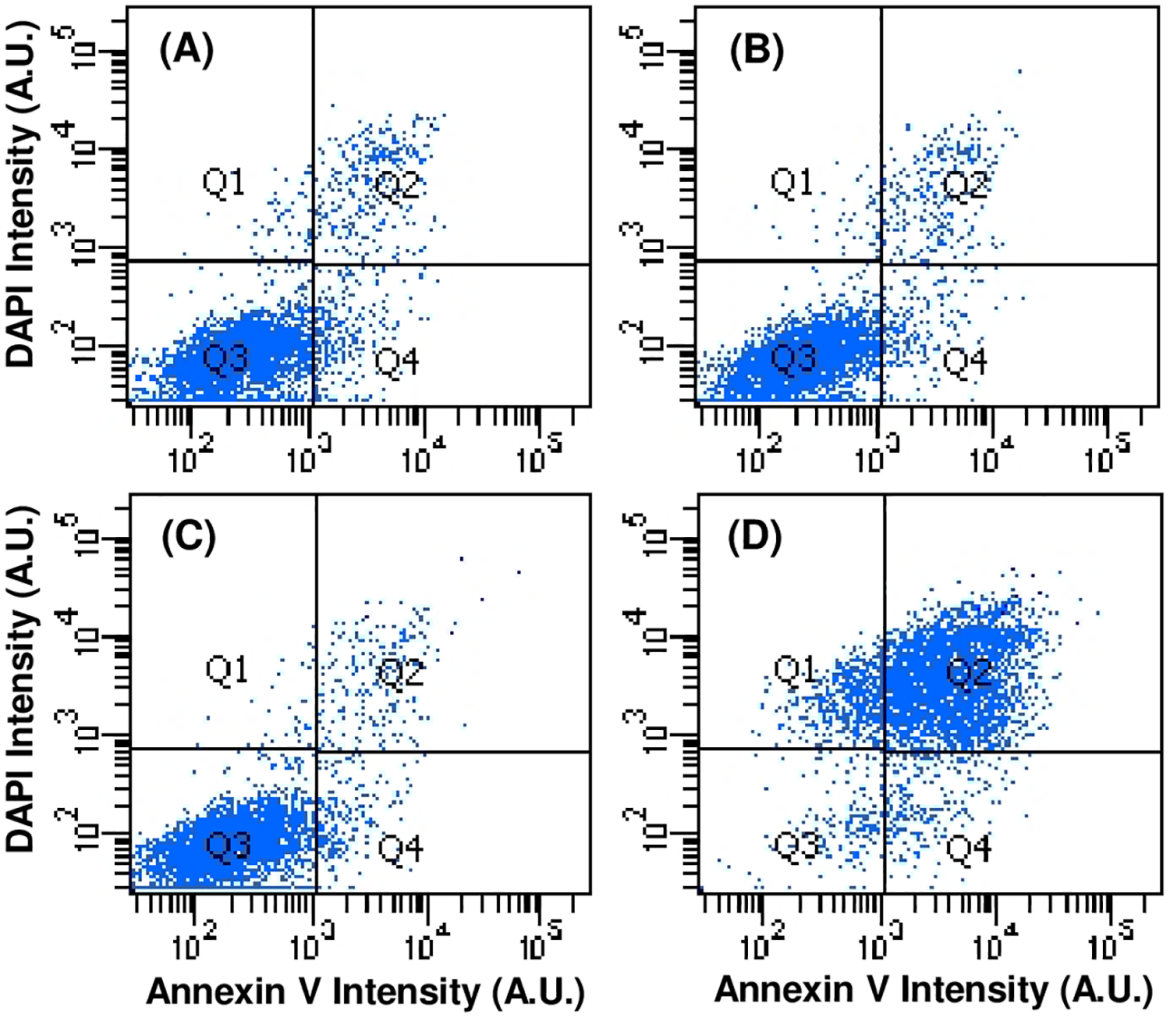
Flow cytometry data for differentiated PC12 cells stained with Annexin V and DAPI. Results are for 24 hrs after exposure to hydrostatic pressure for 3 hrs and then rapid (A) or slow (B) depressurization as compared to a negative control (C: atmospheric pressure) and a positive control (D: 1 μM staurosporine for 24 hrs). Plots are representative of two independent experimental runs.

**Fig 13:**
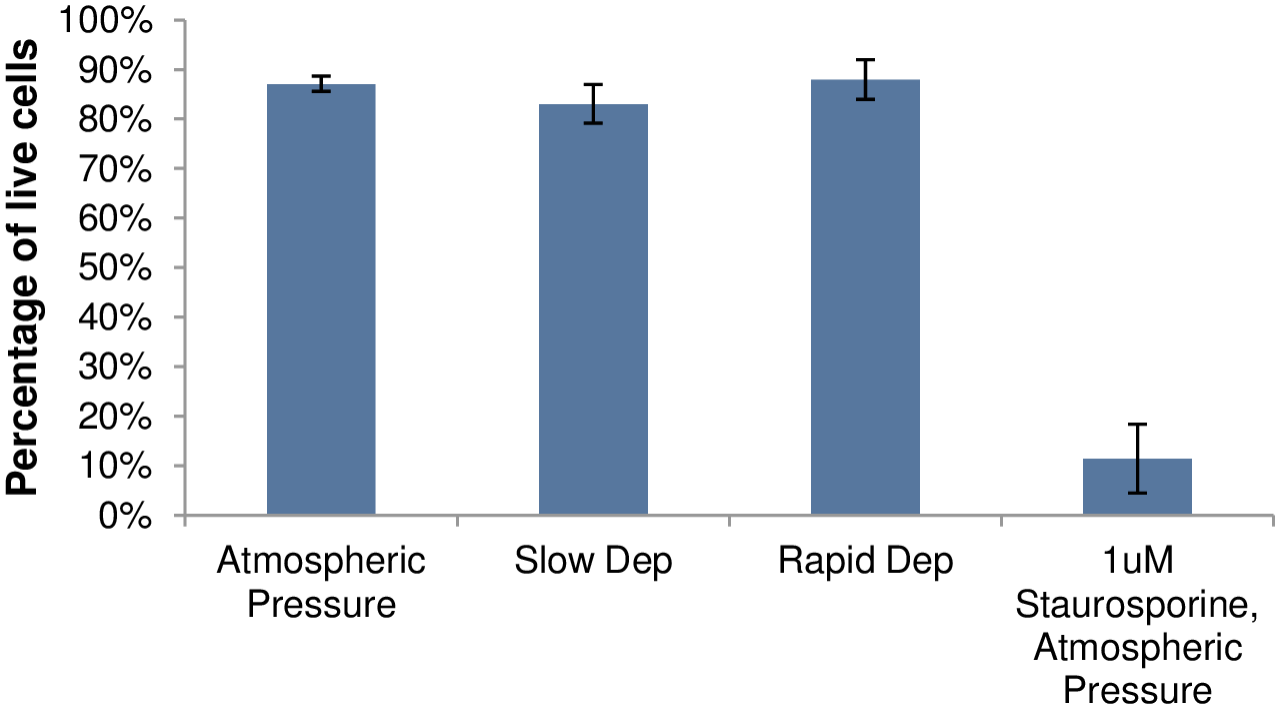
Percentage of viable PC12 cells (Q3 of flow cytometry data show in Fig 12) of cells exposed to elevated hydrostatic pressure for 3 hours and then rapid (A) or slow (B) depressurization as compared to a negative control (C: atmospheric pressure) and a positive control (D: 1 μM staurosporine for 24 hrs). Values represent the means of two independent experimental runs. Error bars represent standard errors.

Annexin V and cell membrane-impermeable DAPI levels were measured in PC12 cells 24 hours after the cells were exposed to 3 hours of hydrostatic pressure and then slowly or rapidly depressurized. In contrast to previous literature [11,26], flow cytometry results (Fig 12) showed no decrease in the percentage of viable cells in either slowly depressurized or rapidly depressurized cells relative to the ambient pressure negative control group (Fig 13, p > 0.5). These data suggest that elevated hydrostatic pressure, regardless of depressurization rate, has no effect on PC12 cell viability or apoptosis levels.

## Discussion

Many studies have reported that following exposure to elevated hydrostatic pressure, changes in cell behavior and function are seen including increased apoptosis, increased proliferation, increased migration, altered levels of certain signal transduction molecules, and changes in cell morphology and cytoskeletal structure [3–5,7,8,10,11,13–23,25,26,28–31,38]. Based on these results, these groups have proposed that hydrostatic pressure could play a significant role in the regulation of homeostatic cell behavior as well as in the development of diseases. These in vitro models have been presented as valuable tools for better understanding disease and identifying new therapeutic targets, for treatment of conditions including glaucoma, cardiovascular disease, and virtually any disorder of physiological systems subjected to physical forces.

However, there has been little or no discussion in these studies on the mechanism by which cells might sense and respond to hydrostatic pressure changes. All known cell mechanosensors are dependent on the induction of mechanical strain within the cell. However, unlike other types of stresses, hydrostatic pressure, at least at the thermodynamically low levels used in these models (10–150 mm Hg), cannot induce mechanical strain in physiological tissues since tissues are essentially incompressible [32]. This has led many to suspect that the reported effects seen in cells exposed to elevated hydrostatic pressure are caused by artefactual changes due to conditions not being carefully controlled rather than by hydrostatic pressure itself [33,35,39,40]. We here explored one such possible artifact that we hypothesized might be responsible for the reported changes in cell behavior, namely the rate of depressurization following application of hydrostatic pressure. Our results did not confirm our hypothesis.

However, our studies also did not confirm a number of the reported changes in cell behavior following application of hydrostatic pressure. As measured by LIVE/DEAD, CellTox, TUNEL, and Annexin V assays, we saw no marked decrease in viability or increase in apoptosis in BAECs following exposure to hydrostatic pressure for time periods of 3 hours to 4 days. In the literature there have been varying reports of the effects of hydrostatic pressure on endothelial cell viability. Although our results are consistent with the findings of some groups [5,15,21], at least two other groups have reported significant drops in endothelial cell viability levels following the application of hydrostatic pressure [2,6]. Cytoskeletal degeneration and some cell death was reported by Tokunaga et al. [2] following 2 to 6 days of exposure to 80 mm Hg or 160 mm Hg compared to an ambient pressure negative control. Thoumine et al. [6] reported a similar loss of BAEC confluency following the application of 40 mm Hg for 24 hrs.

We also did not observe a decrease in viability or an increase in apoptosis in PC12 cells at any time during the 24 hour period following a 3 hour application of elevated hydrostatic pressure (100 mm Hg above atmospheric pressure) as measured by Annexin V and CellTox assays. In contrast, Tök et al. observed increased apoptosis immediately following depressurization for hydrostatic pressure treatments as low as 15 mm Hg lasting for time periods as short as 1 hour [26]. Agar et al. observed increases in PC12 apoptosis levels 24 hours following a 2 hour 100 mm Hg pressure treatment [11]. Our study used similar pressure magnitudes, pressure durations, measurement time points, and measurement assays as these studies, but did not reproduce their results.

We also could not duplicate the reported results on the effects of hydrostatic pressure on BAEC proliferation. We found no increase in BAEC proliferation due to elevated hydrostatic pressure, regardless of depressurization rate, over the course of 1–9 days as measured by two different techniques. In contrast, Sumpio et al. [5] reported statistically significant increases in cell proliferation in BAEC cultures exposed to 40, 80, or 120 mm Hg at times as early as 9, 7, or 3 days respectively. In contrast to our study, Sumpio et al. directly obtained their cells from the intimal surface of the bovine thoracic aorta as opposed to ordering the culture from a third party provider. It is possible that the primary cells they worked with behaved differently than the cell line we used. In addition, Sumpio et al. used collagen coated plates as opposed to the standard tissue culture treated plates used in our study for BAEC culture. Ohashi et al. [21], who cultured BAECs that were purchased from the same supplier as those used in our experiments, observed an increase in the proliferation index of BAECs following an exposure of cells to hydrostatic pressure levels of 50, 100, or 150 mm Hg for 24 hours. Thoumine et al. [6] reported results in contrast with Ohashi et al. [21] by noting a slight decrease in BAEC number following a 24 hour 40 mm Hg hydrostatic pressure treatment on confluent monolayers.

Finally, we did not observe any noticeable changes in BAEC morphology or internal actin filament distribution when exposed to hydrostatic pressure as compared to ambient pressure negative controls, regardless of depressurization rate. Previous studies have reported that exposure to hydrostatic pressure resulted in changes in BAEC morphology and increases in internal actin stress filament concentrations [5,6,15,21]. In some cases, cell elongation [5,15,21] and cellular bilayer or multilayer formation [15,21] were also observed. However, we were unable to replicate these results, and it is notable that a number of the changes reported by some investigators in the literature are inconsistent with those reported by others. For example, reported results ranged from cell elongation [5,21], to cell rounding [6], to a decrease in peripheral actin filament concentration [5,6], to an increase in actin concentration at the periphery [21].

Other groups have also failed to reproduce altered cell behavior following application of hydrostatic pressure. Osborne et al. [40] applied both constant and fluctuating hydrostatic pressures to human organotypic retinal cultures using a gas pressurized chamber. Unlike previous groups [18,38], they did not find any effect of pressure on cell apoptosis levels, integrity of the nuclear layer, or lactate dehydrogenase (LDH) release. Lei et al. [33] found no effect of hydrostatic pressure on astrocyte proliferation, migration, morphology, or cytoskeletal organization.

A study by Ressler et al. [41] noted an upregulation in Egr-1 and TGF-β1 mRNA expression levels within rat tracheal epithelial cells that were subjected to a transmembrane pressure gradient. However, when they applied an elevated hydrostatic pressure of similar magnitude to the cells, no change in mRNA expression levels could be observed. They suggested that the altered gene expression observed under transmembrane pressure was not due to the hydrostatic pressure per se, but by shear and compressive stresses that were induced by the pressure gradient and led to cellular deformation.

While our studies did not show altered behavior of BAEC and PC12 cells following exposure to elevated hydrostatic pressure, the question remains as to why many groups have reported such changes. Lei et al [33] provided a partial answer to this question by showing that in studies using a hydrostatic column to apply hydrostatic pressure to cells, hypoxic conditions are created that alter cell function. However, this does not explain why such changes in cell behavior are also seen in studies in which hydrostatic pressure is applied using a pressurized chamber [2,5,11] or a pump-driven flow system [6,15,21], as such systems do not create hypoxic conditions for the cells (although the former system can generate mildly hyperoxic conditions [35]). Our studies examined the possibility that the rate of depressurization might explain these apparently artefactual findings, but our results were not consistent with that possibility. However, previous experiments performed by Resta et al. [36] did show that rapid pressurizations/depressurizations resulted in blebbing and a loss of membrane integrity in rat retinal ganglion cells. Cell damage increased in severity when multiple rapid depressurization insults were applied within a short time period. In contrast with our studies, Resta et al. applied multiple (up to seven) short bursts (1–7 min in length) of pressure (50 mm Hg) to rat retinas. These bursts were only separated by 1 min intervals [36] as opposed to the more extended periods of pressurization separated by longer intervals that were used in our study. The multiple and rapid applications of pressure and depressurization may explain the differences between their results and ours.

Our study joins a growing body of literature [33,40–42] questioning whether moderate levels of hydrostatic pressure elevation have any effect on living cells. Nonetheless, there are a large number of studies that have reported such effects [2–31]; our results do not explain their findings. It is notable that in none of these studies has a mechanism been proposed to explain how cells that are essentially incompressible can be affected by low levels of hydrostatic pressure elevation. From a medical point of view, there is perhaps a more important question and that is whether there is any evidence that changes in atmospheric pressure on the incompressible tissues of the body have any direct physiological effects. As an example, as far as we are aware, incidence of glaucoma is similar in areas of low average atmospheric pressure (e.g. Denver) as in areas of high atmospheric pressure. This calls into question the basis of such studies.

## Supporting information

S1 Fig CyQUANT dye calibration curve. The calibration curve created for the 24 hr BAEC CyQUANT proliferation experiment using a range of initial cell seeding densities (results shown in Fig 7). The curve relates the intensity of the CyQUANT dye to the number of seeded BAECs.
(PDF)

S2 Fig Additional 24 hr BAEC CyQUANT proliferation experiments using low initial cell seeding densities. Results from two additional 24 hr BAEC CyQUANT proliferation experiments using an initial cell seeding density of 3,125 cells/cm^2^. Note that results from these two experiments could not be aggregated because they were run at different times and CyQUANT dye calibration curves were not created following each experiment.
(PDF)
