# Supplementary material for: The effect of the rate of hydrostatic pressure depressurization on cells in culture
Source: PLoS One. 2018 Jan 9;13(1):e0189890. doi: 10.1371/journal.pone.0189890 (PMC5760025; doi:10.1371/journal.pone.0189890)
Supplement: S1 Fig — The calibration curve created for the 24 hr BAEC CyQUANT proliferation experiment using a range of initial cell seeding densities (results shown in Fig 7). The curve relates the intensity of the CyQUANT dye to the number of seeded BAECs. (PDF) [file pone.0189890.s001.pdf]

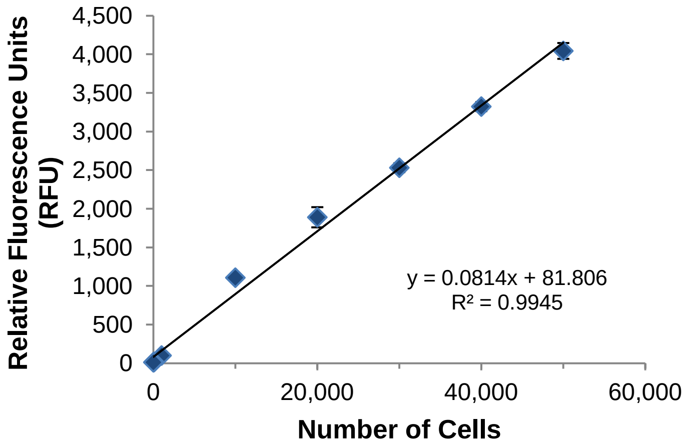

S1 Fig. CyQUANT dye calibration curve. The calibration curve created for the 24 hr BAEC CyQUANT proliferation experiment using a range of initial cell seeding densities (results shown in Fig 7). The curve relates the intensity of the CyQUANT dye to the number of seeded BAECs.
