# Supplementary material for: The effect of the rate of hydrostatic pressure depressurization on cells in culture
Source: PLoS One. 2018 Jan 9;13(1):e0189890. doi: 10.1371/journal.pone.0189890 (PMC5760025; doi:10.1371/journal.pone.0189890)
Supplement: S2 Fig — Results from two additional 24 hr BAEC CyQUANT proliferation experiments using an initial cell seeding density of 3,125 cells/cm2. Note that results from these two experiments could not be aggregated because they were run at different times and CyQUANT dye calibration curves were not created following each experiment. (PDF) [file pone.0189890.s002.pdf]

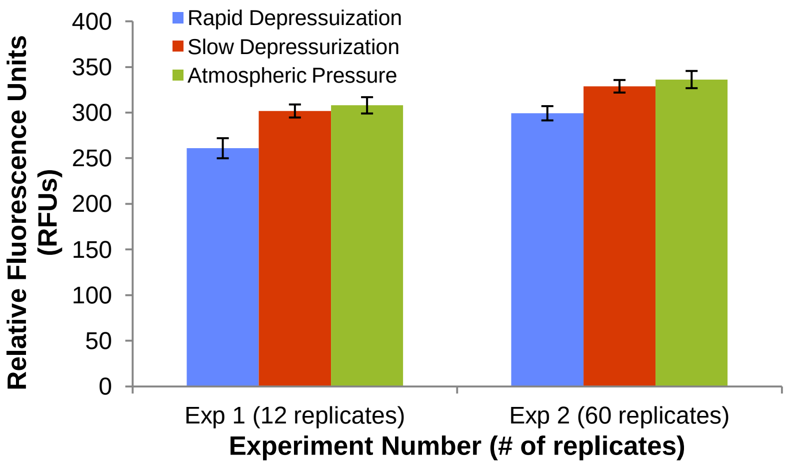

S2 Fig. Additional 24 hr BAEC CyQUANT proliferation experiments using low initial cell seeding densities. Results from two additional 24 hr BAEC CyQUANT proliferation experiments using an initial cell seeding density of 3,125 cells/cm^2. Note that results from these two experiments could not be aggregated because they were run at different times and CyQUANT dye calibration curves were not created following each experiment
